# Observations on Dropsy; Particularly on Ascites Combined with Pregnancy

**Published:** 1824-12

**Authors:** L. Maclean

**Affiliations:** Member of the London Medical Society, and of the Royal Physical Society of Edinburgh.


THE LONDON
### Medical and Physical Journal.

Nº 6 OF VOL. LII.] DECEMBER, 1824. [Nº 310.

For many fortunate discoveries in medicine, and for the detection of numerous errors, the world is indebted to the rapid circulation of Monthly Journals; and there never existed any work, to which the Faculty, in Europe and America, were under deeper obligations, than to the Medical and Physical Journal of London, now forming a long, but an invaluable, series.—RUSH.

### ORIGINAL COMMUNICATIONS,
### SELECT OBSERVATIONS, &c.

ART. I. — *Observations on Dropsy; particularly on Ascites combined with Pregnancy.* By Sir L. MACLEAN, Knt. M.D. (EDINB.); Member of the London Medical Society, and of the Royal Physical Society of Edinburgh.

IN the periscope department of Dr. JOHNSON's excellent Review for September, six cases of ascites during pregnancy, and the treatment adopted, are mentioned. Ascites being at all times an ungovernable disease, and difficult of cure, it is obvious that it must be still more so in such circumstances. The subject, then, is, in every point of view, of paramount importance; since the life of the mother or fœtus in utero, or both, will, in a great measure, depend on the conduct of the practitioner in such cases, as is strikingly exemplified in those alluded to. Of this both the reviewer and reviewed are fully aware, from the solicitude evinced "to have as much information as possible on this obscure point of obstetric surgery."*

I, therefore, readily obey the call on the medical public to contribute such information and facts as may have occurred to their observation, by directing the attention, through the medium of the London Medical and Physical Journal, to a case which fell under my care many years since (1791), referred to by the Editors of this Journal, in their Number for October, and published in the Appendix to my work on Hydrothorax and General Dropsy.†

Though this case excited a considerable degree of interest at the time, yet I am willing to think that, from those reviewed by Dr. Johnson, it may be deemed worthy of greater publicity and attention than it has hitherto had; since it seems to me, and is

* *Medico-Chirurgical Review.*

† We find that a few copies of this work, which was favourably noticed in this Journal at the time of its publication, are still to be had of the publisher, Mr. Nunn, Great Queen-street.—EDITORS.

thought by several medical friends who have perused both, to furnish a complete answer to the information required.

It will appear that, in the subject of this case, Mrs. Constable, ætat. 34, the operation of paracentesis was performed successfully twice prior to, five times during pregnancy, and as many after delivery; that 359 pints of fluid were evacuated; that she went the full time of utero-gestation, without any untoward symptom during or subsequent to these operations, or in her lying-in; that she was delivered of a full-grown male child, which child attained the age of manhood, and is now living and well.

This is the only instance I have met with on record, of the mother and infant surviving the delivery. The former died some months afterwards, from the rupture of an abscess of the right ovarium, which was found, on dissection, to have contained about five pints of pus; and which, it will be admitted, no medical treatment could have averted after I was consulted. But, in my remarks, it is stated, "had this case occurred to me at an earlier period, before impregnation had taken place, and before the stomach had, by sympathy with the uterus, acquired such exquisite susceptibility to medicine, it is probable, from the fortunate results of two cases* which fell under my care the following year, the event might have been favourable." Subsequent experience has fully confirmed this opinion.

At that time the writer of this case was a young physician, without any medical authorities as guides to direct his steps; nor had he ever practised the obstetric art, except in a few instances in the East Indies, from which he could not shrink without giving offence, or leaving the females to nature: yet he determined promptly on the course to be pursued, and acted upon it fearlessly and with confidence of success, having omitted no precaution that was likely to ensure such a result. And here he may be permitted to offer the justly-merited tribute of applause to the respectable surgeon, Mr. Gretton, of Colchester, in attendance with him, who, with a liberality characteristic of real merit, submitted to every hint and suggestion offered, though coming from a junior practitioner.

The plan of treatment adopted in the cases alluded to in the Medico-Chirurgical Review, is so different from that which I would have recommended, and from that practised in Mrs. Constable's case, that I feel a delicacy and difficulty in offering any comments upon it; yet, as the public have an imperative claim upon every medical man's experience, when it is likely to prove beneficial to his fellow-creatures, a few general remarks will not, I trust, be deemed irrelevant or obtrusive.

---

* Cases V. and VI., *Appendix to Inquiry into the Nature, Causes, and Cure of Hydrothorax.*

In expressing my surprise at the advice given by the two highly respectable physicians consulted on Mrs. Langstaff's case, my motive, it is hoped, will not be mistaken. In the first part of the treatment, instead of "general blood-letting, blistering, calomel, digitalis, and squills," resorted to with the view "of relieving the abdominal pain and distention, which were so distressing and urgent as to threaten the life of the patient,"* the immediate evacuation of the water, as the cause, was surely the obvious and only remedy; and to allay uterine action as much as possible, instead of exciting it, "by letting off the liquor amnii."†

But here nature, ever true to her purpose, would not act in obedience to the dictates of art, in contradiction to her own salutary laws: nor, indeed, is it probable that she could, under such inordinate accumulation and distention, until the evacuation of the water brought the parietes of the abdomen in contact with the uterus, and the abdominal muscles thus contributed their powerful aid toward the expulsion of the fœtus. "It will be observed," says the Reviewer, "that, in this and three following cases related, parturition very quickly followed the operation." The cause seems obvious. There can be little doubt that sudden pressure applied to, or removed from, the uterus, would alike derange its functions; and that, if there was a disposition in this organ to contract, from the previous death of the fœtus, the evacuation of the water, for the reasons mentioned, might accelerate abortion. The result in Mrs. Langstaff's case, was such as might be expected: violent symptomatic fever, consequent upon uterine and peritoneal inflammation, demanding the most active remedies to subdue them. The subsequent treatment seems to have been appropriate and judicious.

That these remarks are not the offspring of theory, but the result of what was actually practised with the best effects, a reference to Mrs. Constable's case will show. Before the fourth tapping, a train of alarming symptoms came on from similar causes: excessive distention and pressure, owing to the operation being delayed too long, which immediately ceased on the evacuation of the water. The lady lived ten miles from me, and six from the surgeon, which explains the cause of delay as not attributable to either. The quantity of the water here was greater than at any of the former tappings, being thirty-eight pints.

Such distressing occurrences are not uncommon in ordinary cases of ascites, and though not so serious in their consequences, ought always to be guarded against, when in our power. One instance only shall be mentioned from my Report-book. On

* *Medico-Chirurgical Review.* † *Ib.*

Friday, the 20th December, 1811, I was desired to visit Mrs. Sherriff, a married lady, ætatis fifty-two, residing twelve miles from Sudbury. She had been under medical discipline for several weeks, without receiving any benefit, but had suffered much, for the last few days, from the enormous size and distention of the abdomen. The operation was urgently called for; but not meeting the family practitioner, who, it appeared, had decidedly declared that there was no water in the abdominal cavity, I was compelled, though reluctantly, to postpone my next visit till Monday, the 23d. But early the following morning, my immediate attendance was desired. I found the lady had been suffering under severe abdominal pains and incessant retchings, the greater part of the night. The operation was immediately determined upon, but performed in the most bungling manner, with a tremulous hand; owing, probably, to a secret impression that no water would follow. Water, however, did flow in abundance, though in a slow, irregular, dribbling manner, owing to the cannula not entering the cavity; the stilette having been withdrawn when only the point had penetrated. The operation was irksome and tedious, but the patient was speedily relieved. From this period my respected friend, Mr. Anderson, of this town, attended with me. She was once tapped afterwards by him; and I had the satisfaction of leaving her, on the 10th of February following, perfectly recovered: nor has she had any return since.

On the next (Scarpa's) case, it is only necessary to observe, that a similar oversight was committed, in delaying the operation till the patient "was driven to the point of death;" and that, had this venerable and intelligent surgeon been called in earlier, it is more than probable it would not have been postponed to so late a period. It is necessary, in ordinary circumstances, to draw off the water with great caution, and thus the lives of the twins, as well as that of the mother, might probably have been saved.

In many females, there is a strong predisposition to peritoneal, uterine, and intestinal irritation or inflammation, (especially when the body has been unusually large,) from the sudden collapse succeeding inordinate distention and pressure. How much more, then, are such evils to be apprehended, when ascites has been conjoined with pregnancy. Fully aware of this, every precaution likely to obviate them were observed in Mrs. C.'s case. A regular, uniform, but moderate, pressure was kept up over the whole abdomen, by means of a flannel waistcoat previously fitted to the body, and furnished with straps, to be gradually tightened as the water flowed; and when she was faint, or the motion of the fœtus violent and indicative of uneasiness, a plug was introduced into the cannula till these symptoms subsided. A full dose of laudanum, too, was sometimes previously administered. To these and the other precautions used, the success is to be attributed.

To the third (or Dr. CRUCH's first) case, the same remarks apply, as is very justly observed by the Reviewer. "Drastic purges," it is stated, "and venesection were tried, without affording any relief." Did they do no harm? As I hold the former to be inadmissible in ordinary cases, so in these they must be still more so, by exciting uterine irritation, from sympathy with the intestinal canal; and that the latter was unnecessary, if not injurious, may be inferred from "there being no appearance of phlogosis in the peritoneum, or other part of the abdomen, on dissection." Perhaps it may be said that venesection prevented this. "But the operation gave immediate relief:" this speaks for itself.

In the remaining three cases, the serious consequences of delay, and most probably of the neglect of proper precautions during the operation, are obvious.

In the last case but one, which was combined with universal anasarca to an alarming degree, it is highly probable that acupuncture, in preference to scarifications, would have proved a valuable and powerful auxiliary. I am induced with the more confidence to recommend it, from the great relief and benefit derived from it in a case which has recently occurred to me.

Samuel Cooper, a butcher, ætatis thirty-nine, after suffering for some time under hepatic and visceral disease, from a long course of intemperance, became dropsical in May last. He had ascites and anasarcous swellings of the lower extremities, up to the hips. After taking a few medicines from Mr. Lewis, a highly respectable practitioner of this town, he was advised to go to London, and was admitted into St. Bartholomew's Hospital about the 12th or middle of June. He remained there till the 15th July, when his friends wished him to return into the country; his disorder having progressively advanced, and his general health having suffered from confinement. Tapping was proposed the following week, had he remained. Besides other remedies, drastic purgatives, by his account, were occasionally prescribed. He came to me on the 17th July. The whole abdomen, the lower extremities, the hips, even the lumbar region, were enormously distended with water. The countenance was cadaverous; the conjunctiva of a deep yellow hue; the breathing extremely difficult, threatening suffocation on motion. Some parts of the abdomen were of a livid colour, and the veins distended with dark blood. I advised immediate tapping, with the precautions already mentioned, though I dreaded the result; and prescribed strong bitters of the infus. gent. c. and calumba, in combination with saline diuretics ter die ; the compound squill pill and calomel every night. He was tapped on the 21st, and four gallons of fluid drawn off; but, though this was done very slowly and with great judgment, he had nearly sunk under the operation. The anasarcous swellings quickly disappeared ; but the body swelled in proportion, so as in a few days to have attained nearly its original size, and so as to require tapping again on the 28th ; when the same quantity of fluid was evacuated. He, however, bore the second operation much better than the first.

The body has not swelled since ; but, as he began to walk about, the legs became again anasarcous ; with the view of relieving which, acupuncture was advised. This he amused himself by performing on different parts of the feet, legs, and thighs, by which a regular and copious discharge was kept up for some weeks ; and, as the punctures healed, which was generally in two or three days, fresh ones were made.

On the 18th September, I saw him for the first time since the 17th July, having been from home when he called at different times. No water in the abdomen, and but little œdema of the feet and ankles towards evening. He had desisted from the acupuncture for ten days ; had gained flesh and strength ; no yellowness of the conjunctiva ; urine in full quantity, bowels lax ; mouth soon became affected, and he has been kept under the influence of the mercury since. The medicines were varied as circumstances indicated ; and, together with the tonic and diuretic mixture, he has been of late taking a strong infusion of the broom *(genista)*, with the supertartrated potass, the squill, and blue-pill, h. s.

September 25.—Continues to improve, and considers himself recovered. Urine clear, and voiding from four and a half to five pints daily.

Should the above abstract meet the eye of Dr. Hue, under whose care the patient was, he will recollect the case, and be surprised, but gratified, at the result.

In the course of a tolerably extensive practice of thirty-four years in this country, many cases of ascites, both simple and combined with general dropsy, as well as during pregnancy, have fallen under my care, and I have had every reason to be satisfied with the treatment adopted. My first object has been, and I think ought always to be, to ascertain what organic derangement, if any, existed as a cause? whether hepatic, splenetic, ovarian, or mesenteric ? or whether the water was contained in a sac, or in the abdominal cavity, or both, which sometimes happens ? Having formed as accurate a judgment of the whole case as circumstances permitted, the indications of cure were regulated accordingly. A course of saline, mild, aperient diuretics, in combination with the bitter infusions and tinctures, squills and mercury at bed-time, steadily persevered in for a length of time, till the object in view be accomplished, or till every prospect of removing the water by the natural outlets be cut off, will be found the safest and most effectual practice. Mercury in the mildest form, and in moderate doses, should be carried to the extent of affecting the salivary glands, and the habit kept as long as may be thought necessary under its influence. Without these precautions in the cases for which it is peculiarly adapted, there will be no security from returns; and it may be worthy of remark here, that they are the opposite to those in which digitalis exerts its salutary influence, as has been long since observed elsewhere. "If the disease occur in delicate subjects of either sex, endowed with a high degree of susceptibility, with a pallid countenance, a soft, smooth, thin skin; a white or transparent appearance of the anasarcous swellings, readily pitting on pressure, and retaining the impression of the finger; more especially in females, from frequent parturition, profuse uterine or other hemorrhages, or similar debilitating causes, without any organic affection,—the digitalis alone will generally succeed in evacuating the water: but its salutary effects will be greatly promoted by mild tonics, steel in particular, and moderate doses of the fixed vegetable alkaline salts. Calomel, squills, and the crystals of tartar, if at all admissible, must be administered with extreme caution, and in very small doses."* Digitalis ought never to be trusted to alone in any case, and in simple ascites I have found it of no use, though repeatedly tried; but when combined with anasarcous swellings, of the character above described, it will prove a good auxiliary. When there is no pregnancy, frictions to the whole abdomen, with the liniment. hydr. or the ung. hydr. saturated with camphor, and as tight a bandage as can be borne, removed at bed-time, will contribute materially to the cure.

The practitioner must be satisfied with a slow and gradual improvement; for, if his impatience prompt him to the use of drastic purgatives, or other violent remedies, as is too often the case, with the view of accomplishing a cure speedily, he will be greatly disappointed. His object should be to evacuate the water *per vias urinas*, as recommended by Baglivi, Riverius, and other respectable writers, and amply confirmed by my own experience: for though such harsh means, as advised by some, may occasionally afford temporary relief, and carry off much watery fluid by the bowels, yet, by exhausting the vital powers, they for the most part confirm the disease, and the water will accumulate more rapidly afterwards. By pursuing a different

---

* *Inquiry into the Nature, Causes, and Cure of Hydrothorax and general Dropsy,* p 163 et seq.

course, the general health and strength will be improved ; and, should the operation eventually be necessary, the habit will be in a more favourable state for it, the kidneys more readily acted upon, and the chance of recovery proportionably increased.

As far as regards bleeding, though it cannot be denied that instances of dropsy will sometimes occur in which free and copious depletion may be indicated, and even indispensable, before we can hope to make any impression upon the absorbents or kidneys, yet my experience fully warrants the assertion, that they are comparatively rare, and that it is not to be numbered among the general remedies in this disease. When the pulse is tense and cordy, the anasarcous swellings at the same time are hard and resisting, the complexion florid, with symptoms of general plethora, the bodily strength not much impaired, the disease has been of short duration, and the subject is in the prime of life, the tone of the arterial system must be relaxed and subdued by venesection, before digitalis or other diuretics can be expected to produce their salutary effects.* The excellent case of Professor GRAHAM, of Edinburgh,† affords a striking illustration of this; and, had I seen many of my patients at an earlier period, a similar plan would most probably have been adopted, since in some of them the serous effusion was doubtless the consequence of the neglect of venesection in the early stages ; but in others to the too free and injudicious use of the lancet, especially in the advanced stages of inflammatory affections. But dropsy in general, as it has fallen under my care, I have found to be a disease of exhaustion and debility, occasionally, however, accompanied with local congestions of blood ; especially in hydrothorax, owing to the interruption to pulmonary circulation from pressure, which speedily gave way on the absorbents and secreting organs being freely acted upon, or on the cause being removed, without the necessity of blood-letting. In hospital practice, the case may be different, where the patients are admitted immediately on, or soon after, the attack ; but in the country, where they have probably been for weeks under other discipline before the physician is consulted, it is far otherwise.

While on this part of the subject, I cannot help adverting to the practice of our continental medical brethren. We owe so much to them for their researches and discoveries in physiology, pathology, and chemistry, that we ought to speak with reserve of their treatment of diseases ; but, in some of the most acute and inflammatory affections, their practice seems to me to be

* The kidneys and bowels will sometimes act spontaneously in such circumstances, though previously the most powerful diuretics and purgatives made no impression upon either. *Vide infra*, p. 26.

† *Edinburgh Med. and Surg. Journal*, No. 71, p. 226.

worse than inert and useless; for many of them trust to the abstraction of a few ounces of blood by cupping or leeching, or mild aperient ptisans; while in other instances they go into the opposite extreme, as the cases under review demonstrate: and though their public hospitals, and other similar institutions, furnish opportunities of post-mortem examinations, which we in this country do not possess, yet they seem not to profit by them, but proceed in the same inefficient course; of which our medical Journals furnish many examples from their writings.

Where blood-letting is imperatively called for, it should be prompt, free, and quickly repeated, until the inflammatory action be subdued. I have for many years inculcated the maxim, "not to look at the quantity of blood drawn, but at the effect," and have not been satisfied to carry it *ad deliquium;* but, after directing the arm to be untied, have waited till the patient recovered, and if the disease was not subdued, or the strength of the pulse not materially and permanently reduced, have again and again had the ligature re-applied, and proceeded till the end in view was obtained: and I have the satisfaction in reflecting that many lives were thus saved, which would otherwise have been sacrificed.

The cases most embarrassing to young, and even to experienced practitioners, are those which are so masked by distant sympathies of vital organs, that the pulse is often extremely quick, feeble and small, fluttering, irregular, or intermitting, yet where the inflammation is making serious and rapid progress in some organ, not yet suspected, because no pain is, perhaps, complained of there. In such doubtful instances, where venesection was not previously thought of, or was condemned as improper, my maxim has been—"let us look at the blood," and desist after the loss of a few ounces, if found ineligible, or proceed, if otherwise; and be guided in the quantity by the effect, and in some degree by its appearance. How often have I had the gratification of observing the pulse become slower, full, and regular, respiration free and easy, the cough less urgent, the expectoration loose and copious, in such circumstances, after the heart and vascular system were relieved of the accumulated load. Such cases are very common, but often overlooked or mistaken, until it be too late.

While on this subject, one case shall be mentioned, where a plan nearly similar to that adopted by Dr. Maxwell, of Dumfries, recorded in the Edinburgh Medical Journal, was followed, and with equal success. The disease was insidious and masked at first, yet I had no hesitation in pronouncing it that species of acute phrenitic inflammation preceding watery effusion in the head, improperly termed hydrocephalus acutus; and this opinion was confirmed by the information received, that a brother of the patient had died of this disease. Mr. Corder, near Coggleshall, Essex, was the subject of this case: he was of a nervous, timid disposition. The pulse varied during the examination from 76 to 120 in a minute, was small, but cordy. Finding that he fainted when only a few ounces of blood were drawn before my visit, I directed him to be bled in a recumbent posture, and, when syncope came on, the ligature to be untied until he recovered, when it was again applied. This was done repeatedly during, and in the intervals between, my visits; for he resided sixteen miles from me. Leeches, blisters, and other active means, were at the same time resorted to; but I am convinced that without venesection, quickly repeated in the manner stated, they would have proved of no avail; and that it is the only remedy to be trusted to in limine, with any prospect of success. An interesting feature in this case was, that the bowels acted spontaneously during the almost total suspension of the circulation, while before, the most drastic purgatives had little effect; and though mercury was used constantly, both externally and internally, it had no effect on the salivary glands, until venesection was carried to the utmost extent, and was no longer necessary or admissible. I was called to this patient on the 13th, and took my leave on the 26th September, 1819. He has enjoyed good health since.

Having insensibly extended this communication far beyond the limits intended, it is full time to bring it to a close. I would only further beg leave to add a few words on the pathology of dropsy. From the foregoing observations, it is not likely that I should subscribe to the modern pathology of this disease, though it has so able an advocate as the late excellent Dr. Parry among its supporters. This doctrine has always appeared to me to be founded on a partial, and consequently erroneous, view of the real state of things. Experience does not sanction the opinion of the universality of the buffy coat of the blood of dropsical subjects; and, even if it did always or generally exist, it has been clearly ascertained that it is not an unequivocal test of inflammation. That dropsy is sometimes preceded by, or accompanied with, inflammation, or increased action of the heart and corresponding momentum of the blood, no one will deny, as has already been adverted to; but, that by far the greater number of dropsical effusions—I would say nine in ten, are the result of exhausting and debilitating causes, without any traces of previous inflammatory action, any one who has carefully and impartially investigated the subject will, I think, admit. Dr. Parry seems to allow that this is sometimes the case, but infers, though he has not proved, the existence of previous inflammatory excitement. The doctrine is the more dangerous, as being advocated by so able a pathologist and physician, because it leads to a most dangerous practice, if generally acted upon. Still less can I assent to the opinion advanced by him, that the absorbents are not affected or concerned in dropsy, though his able reviewer is a convert to it.* To admit this, would be to abandon one of the first indications in the cure.†

As to the coagulability or non-coagulability of dropsical urine by heat, I have never tried, nor am I ever likely to try it, because I always regarded it as a vague and inconclusive test; and I believe it has been amply proved to be so by others. Even if it were to be depended upon, it could not be adopted in general,—certainly not in country practice.

*Sudbury; September 26th,* 1824.

* *Medico-Chirurgical Review,* No. ix. p. 13.

† *Inquiry into the Nature, Causes, and Cure of Hydrothorax,* passim.

